# Biomechanical comparison of meniscus-suture constructs for pullout repair of medial meniscus posterior root tears

**DOI:** 10.1186/s40634-019-0186-4

**Published:** 2019-04-15

**Authors:** Shinichiro Okimura, Tatsuo Mae, Yuta Tachibana, Ryo Iuchi, Ken Nakata, Toshihiko Yamashita, Konsei Shino

**Affiliations:** 1 Department of Orthopaedic Surgery, Sapporo Medical University School of Medicine, South-1, West 16, Chuo-ku, Sapporo, Hokkaido 060-8543 Japan; 2 Department of Orthopaedic Surgery, Osaka University Graduate School of Medicine, 2-2 Yamada-oka, Suita, Osaka, 565-0871 Japan; 3 Sports Orthopaedic Center, Yukioka Hospital, 2-2-3 Ukita, Kita-ku, Osaka, Osaka 530-0021 Japan; 4 Medicine for Sports and Performing Arts, Department of health and Sports Science, Osaka University Graduate School of Medicine, 2-2 Yamada-oka, Suita, Osaka, 565-0871 Japan

## Abstract

**Background:**

Transtibial pullout repair for posterior meniscus root tear is widely performed to restore meniscal function. However, it is sometimes technically difficult to pass the suture through the posterior medial meniscus root in narrow joint space. To address this limitation, a new suture technique using an all-inside meniscal suture device through the tibial tunnel was proposed. The purpose of the present study was to compare the biomechanical properties of a meniscus-suture construct prepared using an all-inside meniscal suture device and those of the construct prepared using conventional suture techniques.

**Methods:**

A total of 18 fresh-frozen porcine medial menisci were used and randomly divided into three groups according to the type of suturing technique applied. Three suturing methods were evaluated: suturing with all-inside meniscal suture device, single simple suture, and double simple sutures. All specimens were subjected to cyclic loading of 300 cycles followed by a load-to-failure test. The displacement after cyclic loading, the ultimate failure load, and the mode of failure were evaluated.

**Results:**

There was no significant difference among the three suturing techniques regarding both displacement after cyclic loading and ultimate failure load. Suture breakage was the most common failure mode in each group.

**Conclusions:**

The biomechanical properties of meniscus-suture construct with the all-inside meniscal suture device were equivalent to those obtained using conventional suture techniques. Our results suggest that pullout repair using the all-inside meniscal suture device through the tibial tunnel could serve as an alternative suture technique for the repair of posterior meniscus root tears.

## Background

The meniscus plays an important role in load distribution and joint stability. To maintain a healthy articular cartilage, it is crucial to maintain the hoop strain mechanism and circumferential integrity of the meniscus. Once a medial meniscus root tear occurs, the contact pressure in the medial compartment increases to the level similar to that observed following total meniscectomy (Allaire et al., 2008; Padalecki et al., 2014). Thus, meniscus root tears should be repaired anatomically to preserve meniscal function and prevent subsequent cartilage degeneration (Johannsen et al., 2012).

Meniscus root repair is widely performed to restore meniscal function (Feucht et al., 2015a; LaPrade & LaPrade, 2014; LaPrade et al., 2017; Lee et al., 2014a). While both transtibial pullout repair and suture anchor repair are well-established techniques for the management of posterior meniscus root tears, transtibial pullout repair is more commonly used and has better clinical outcomes (Ahn et al., 2007; Lee et al., 2014b; Petersen et al., 2014). However, it is sometimes technically demanding to pass the suture through the posterior medial meniscus root in a narrow joint space. To address this limitation, we propose a new suture technique that involves using an all-inside meniscal suture device through the tibial tunnel. First, the tibial tunnel is created in the same fashion as for the conventional pullout repair. Then, the all-inside meniscal suture device is inserted into the meniscus through the tibial tunnel, and repair is conducted entirely within the joint space. While this novel technique is advantageous in that it enables repairing the meniscus even if the joint space is narrow, the biomechanical properties of this suture method remains unclear.

In the present study, we aimed to compare the biomechanical properties of meniscus-suture constructs using an all-inside meniscal suture device against those obtained using conventional suture techniques. We hypothesized that the mechanical properties of meniscus-suture constructs using an all-inside meniscal suture device would be similar to those using conventional suture techniques.

## Methods

### Study design

In this study, we used porcine menisci to evaluate the mechanical properties of meniscus-suture construct, which represents a well-established practice in biomechanical research (Feucht et al., 2015b; Forkel et al., 2017). This study was approved by our institution’s internal review board. All applicable international, national, and institutional guidelines for the care and use of animals were followed.

### Specimen preparation

A total of 18 fresh-frozen porcine medial menisci were used. The mean age of the pigs at the time the specimens were obtained was 24 weeks (age range, 23–25 weeks). All specimens were kept frozen at − 20 °C and thawed at 4 °C for 24 h in a refrigerator before biomechanical testing. After removing the patella, patellar tendon, muscles, and ligaments, the medial meniscus was sharply resected from the bony attachment to the tibia at the anterior and posterior root with a scalpel and completely detached from the tibial insertion. The specimens were randomly divided into three groups (six specimens per group) according to the type of suture technique applied.

### Suturing technique

A No. 2 polyester suture was whip-stitched to the anterior horn of each medial meniscus. Three suturing techniques for the medial meniscus posterior root were tested. To minimize the effect of other factors on the comparison of suturing techniques, we used the same suture materials and the same number of suture strands to pull the meniscus in each specimen.

**Fig. 1:**
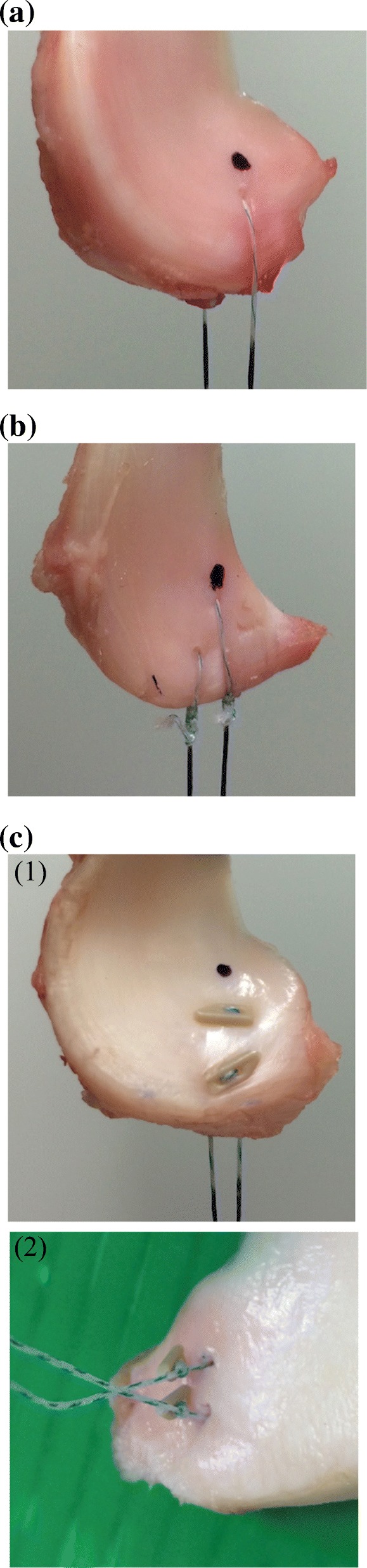
Suture techniques. (**a**) Single simple suture group. A 2–0 Ultrabraid suture was passed through the medial meniscus posterior horn 5-mm away from the posterior edge. (**b**) Double simple sutures group. Two separate 2–0 Ultrabraid sutures were passed through the medial meniscus posterior horn 5 mm away from the posterior edge of the specimen, with a distance of 5 mm between the two sutures. (**c**) All-inside meniscal suture device group (1) Superior side. The first PEEK anchors of the two FasT-Fix 360® devices were installed through the medial meniscus posterior horn 5-mm away from the posterior edge, with a distance of 5 mm between the two anchors. (2) Inferior side. Before removal of remaining anchors

Six specimens were sutured using the single simple suture technique (single simple suture group), according to the following protocol: one 2–0 Ultra High Molecular Weight Polyethylene suture (Ultrabraid®, Smith & Nephew Endoscopy, Andover, MA, USA) was passed through the medial meniscus posterior horn 5-mm away from the posterior edge of the specimen (LaPrade et al., 2015a) (Fig. 1a).

Another six specimens were sutured at the medial meniscus posterior horn using the double simple suture technique (double simple suture group), according to the following protocol: two separate 2–0 Ultrabraid® sutures were passed through the medial meniscus posterior horn 5 mm away from the posterior edge of the specimen, with a distance of 5 mm between the two sutures. Each suture was fastened and one end of each suture was cut to use two strands in the testing, while all four strands were used in the double simple suture in the previous study (Rosslenbroich et al., 2013) (Fig. 1b).

The remaining six specimens were sutured at the medial meniscus posterior horn using two all-inside meniscal suture device with 2–0 Ultrabraid® sutures (FasT-Fix 360®; Smith & Nephew Endoscopy, Andover, MA, USA) (all-inside suture device group), according to the following protocol: the first polyether ether ketone (PEEK) anchors of the two FasT-Fix 360® devices were installed through the medial meniscus posterior horn 5-mm away from the posterior edge of the specimen, with a distance of 5 mm between the two anchors, while both of the remaining anchors were cut and removed (Fig. 1c). We used the two FasT-Fix 360® devices to match the number of strands in the single simple suture and double simple suture groups.

### Biomechanical testing

**Fig. 2:**
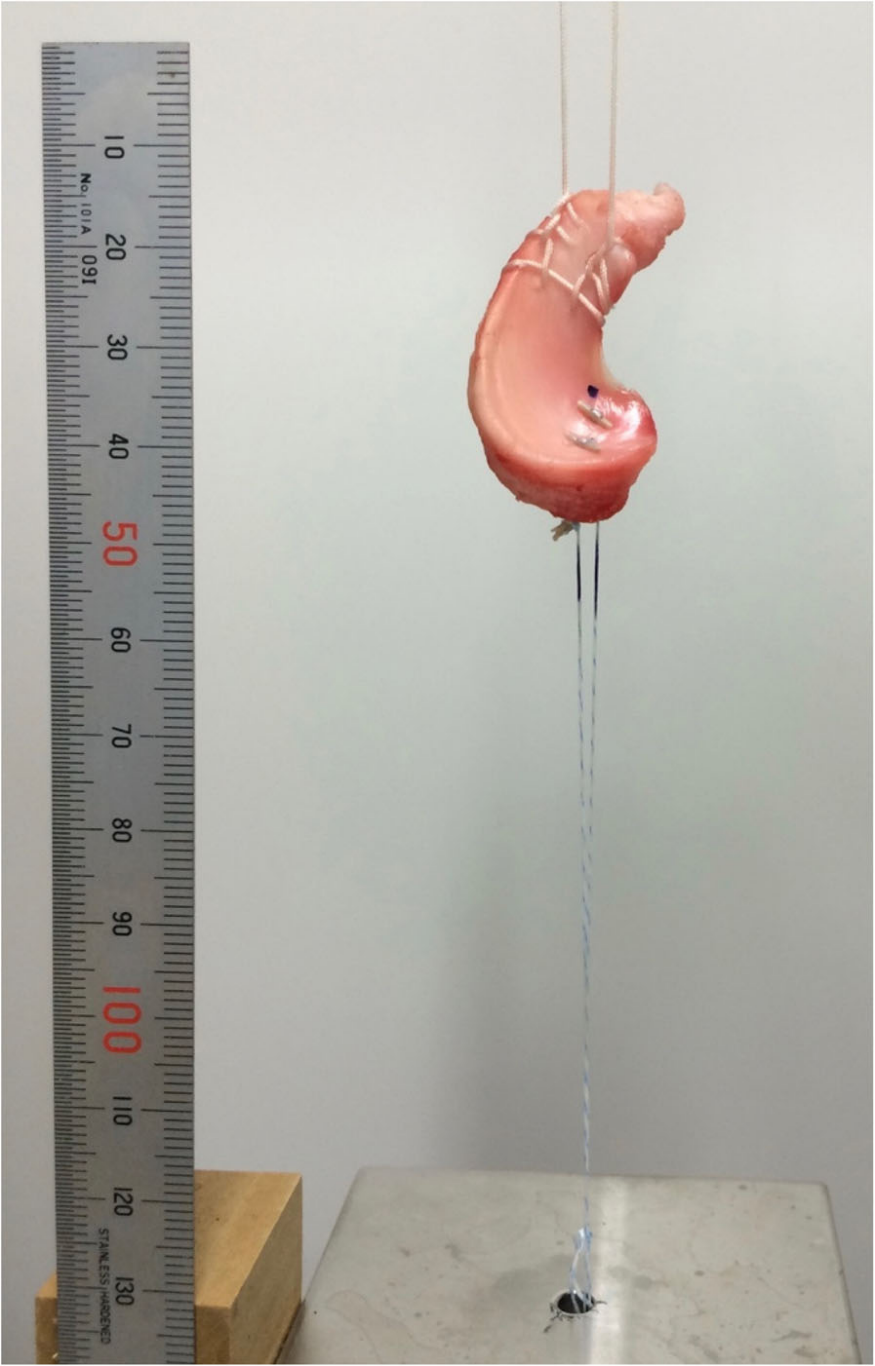
Set-up for biomechanical testing. The sutures stitched the anterior horn were fastened to the upper clamp. The sutures introduced to the posterior horn were tightened over the metal jig

Biomechanical testing was conducted on a material testing machine (AUTOGRAPH AG-IS; SHIMADZU, Kyoto, Japan). The sutures stitched the anterior horn were fastened to the upper clamp. The sutures introduced to the posterior horn were tightened over the metal jig with Endobutton® (Smith & Nephew Endoscopy, Andover, MA, USA) (Fig. 2).

**Fig. 3:**
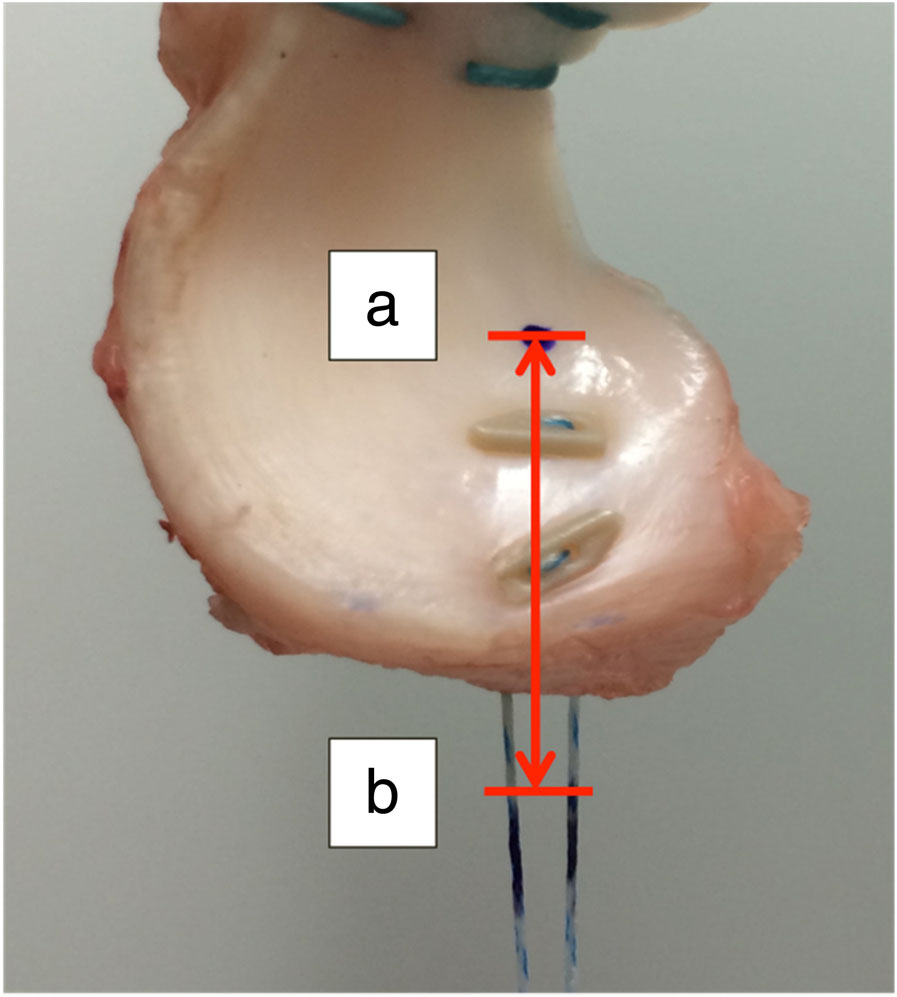
Measurement of displacement. One dot was marked on the meniscus (**a**). The reference point was fixed at 10 mm below the meniscus edge (**b**). The distance from the reference point to a small dot marked 3 mm anteriorly to the suture or anchor was measured

One dot was marked on the meniscus and the other was marked on the suture introduced to the posterior horn. Afterwards, a cyclic loading test was performed at a loading force between 5 and 20 N and a rate of 200 mm/min for 300 cycles. Following the cyclic loading test, a load-to-failure test was performed at a rate of 5 mm/min. These procedures were recorded using a video recorder (HDR-CX370V; SONY, Tokyo, Japan). Instead of measuring the distance between the clamps, all measurements were performed using image analysis software (DIPP Motion Pro 2D; DITECT, Tokyo, Japan). This software automatically tracks and captures the dot. It can measure the distance between the dot marked on the meniscus and the reference line. Thus, we could measure the actual displacement caused by loading, excluding the effect of slippage between clamps as well as the stress-relaxation response of the soft tissue. For image analysis, the reference point was fixed at 10 mm below the meniscus edge at the display of the software and we measured the distance from the reference point to a small dot marked 3 mm anteriorly to the suture or anchor (Fig. 3). The displacement caused by a loading force of 5 N was defined as the difference between the distance measured at the first cycle and the distance measured at the last cycle. Additionally, the ultimate failure load and the mode of failure were recorded.

### Statistical analysis

One-way analysis of variance (ANOVA) was performed to compare the overall effect of suture technique on displacement and ultimate failure load, followed by Tukey’s post-hoc test to detect significant differences. The Fisher’s exact test was used to evaluate the differences in mode of failure. *P*-values of less than 0.05 were considered to indicate statistical significance. Power analysis indicated that the minimum sample size necessary to detect a meaningful difference among three independent groups was six menisci per group.

## Results

### Displacement after cyclic loading

No suture breakage was observed during a cyclic loading test. There was no significant difference among the three suture techniques in terms of displacement after cyclic loading (Table 1).

**Table 1:** Displacement after cyclic load and ultimate failure load

Group	Displacement after cyclic loading	Ultimate failure load
	(mm)	(N)
Single simple suture group	Mean 0.76 (SD 0.68)	Mean 111.6 (SD 12.6)
Double simple sutures group	Mean 1.17 (SD 1.08)	Mean 107.2 (SD 10.3)
All-inside meniscus suture group	Mean 0.75 (SD 0.66)	Mean 96.9 (SD 16.2)

There was no significant difference among three groups

### Ultimate failure load

There was no significant difference among the three techniques regarding the ultimate failure load (Table 1).

### Mode of failure

Suture breakage was observed in four specimens of the all-inside suture device and single simple suture groups as well as in five specimens of the double simple suture group. One specimen in the all-inside suture device group failed because of anchor pullout (Table 2). All the suture breakages occurred on the inner sutures in both the all-inside suture device and double simple suture groups. There was no significant difference among the three suture techniques regarding the mode of failure.

**Table 2:** Mode of failure

Group	Suture breakage	Meniscus cut out	Anchor pulling out
SS	4	2	
DS	5	1	
AI	4	1	1

There was no significant difference among three groups

## Discussion

The principal finding of the present study is that the biomechanical properties of meniscus-suture construct obtained using the all-inside meniscal suture device were equivalent to those obtained using the single simple suture and double simple suture techniques. Thus, the results of our study suggest that the pullout repair of the posterior meniscus root tear with the all-inside meniscal suture device through the tibial tunnel can serve as an alternative to the repair performed with conventional suture techniques.

The maximum failure load of the native medial meniscus posterior root has been reported at 359–678 N (Ellman et al., 2014; Kopf et al., 2011; Mitchell et al., 2016). Meniscal root repair with insufficient strength of meniscus-suture construct might result in failure before completion of the healing. Stärke et al. (Starke et al., 2013) clarified the tensile forces acting on the repaired medial meniscus posterior root under different loading conditions in cadaveric knees, reporting the highest mean tensile force at 60.1 N. Similarly, Kim and Joo (Kim & Joo, 2013) reported a mean pullout failure force of 71.6 N for simple vertical suture introduced to the medial meniscus posterior horn. In this context, one may conclude that no suture technique described in the present or previous studies can restore the native strength of the medial meniscus posterior root (Ellman et al., 2014). However, such biomechanical studies evaluated only time-zero stability (i.e., immediately after repair, before completion of the healing), and the minimal strength of the meniscus-suture construct required to ensure stability of the repaired medial meniscus posterior root. The meniscal healing process is likely affected not only by biomechanical factors related to the repair but also by biological factors intrinsic to the meniscus. Therefore, the repaired meniscal root may acquire enough fixation strength after completion of the healing if sufficient initial stabilization has been achieved.

Several suture techniques have been developed for the repair of the posterior meniscus root tear. Feucht et al. (Feucht et al., 2013) compared the biomechanical properties of meniscus-suture constructs among four different suture techniques currently used for transtibial pullout repair of posterior meniscus root tears. In this study, the modified Mason-Allen suture technique provided superior biomechanical properties of the meniscus-suture construct compared to those using double simple suture or horizontal mattress suture. On the other hand, LaPrade et al. (LaPrade et al., 2015b) reported that the modified Mason-Allen suture and the double simple suture were not significantly different in terms of the biomechanical properties. However, the authors admitted that the modified Mason-Allen suture is more technically demanding, and suggested that the simple suture technique might represent the clinical standard for posterior meniscus root repair because of its technical simplicity and acceptable biomechanical outcomes. Therefore, we compared the biomechanical properties of single or double simple suture and that of the all-inside meniscal suture device.

Rosslenbroich et al. (Rosslenbroich et al., 2013) compared the biomechanical properties of transtibial pullout repair using either single or double simple suture, and reported that ultimate failure load of the double suture was almost twice as high as that of the single suture. However, this previous study employed two strands for single simple suture and four strands for double simple sutures, whereas we compared three techniques employing the same number of sutures and the same suture materials, finding no significant difference regarding the ultimate failure load. Our results thus describe the effect of the suture technique itself. The discrepancy between our results and those of Rosslenbroich’s study may be due to the difference of the biomechanical properties of the suture materials themselves, suggesting that stronger suture materials may contribute to achieving superior mechanical properties of the repair.

During the load-to-failure test, most specimens in the all-inside suture device and double simple suture groups exhibited suture breakage but the two sutures were never broken. This may suggest that there exists an imbalance between the two strands, as the fiber arrangement of the meniscus was not parallel to the direction of the tensile force. In our study, the inner sutures always failed before the outer sutures, indicating that the inner suture was exposed to higher loads.

The present study has several limitations. First, we did not use the bone tunnel model, whereby the button-bone interface regulates loading displacement of the meniscus-suture complex in the repair construct (Cerminara et al., 2014). We just modified the bone tunnel model to use a metal fixation device for minimizing the deformity. Thus, we could evaluate the meniscus-suture interface directly. Second, we did not apply the transtibial technique in this study. This was done to arrange the condition of meniscus-suture constructs of each specimen and accurately evaluate the biomechanical properties as they have been in previous studies (Kopf et al., 2011; LaPrade et al., 2015b). Third, the direction of the suture pull was not physiological. Our biomechanical testing protocol was designed to simulate a worst-case scenario, in which the load to the meniscus was applied parallel to the meniscal fibers. This might not reflect in vivo loading of the meniscus, which also involves shear and compressive forces. However, we believe that this model is useful because it allowed us to evaluate the influence of the suture method independently from that of other factors. Fourth, the porcine meniscus was used instead of a human meniscus because most human cadavers available to us came from elderly donors and thus likely exhibited degenerative menisci; under these circumstances, using the menisci of adult, healthy pigs were preferable because it reduced the variation in biomechanical properties across different specimens, allowing us to focus on the effect of the suture technique itself.

In this porcine study, we found that the biomechanical properties of meniscus-suture construct with the all-inside meniscal suture device were equivalent to those with conventional suture techniques. Our results suggest that pullout repair using the all-inside meniscal suture device through the tibial tunnel could serve as an alternative suture technique for the repair of the posterior meniscus root.
